# Quantum interference based Boolean gates in dangling bond loops on Si(100):H surfaces

**DOI:** 10.1038/srep14136

**Published:** 2015-09-15

**Authors:** Andrii Kleshchonok, Rafael Gutierrez, Christian Joachim, Gianaurelio Cuniberti

**Affiliations:** 1 Institute for Materials Science, Dresden University of Technology, TU Dresden, 01062 Dresden, Germany; 2 GNS & MANA Satellite, CEMES-CNRS, 29 rue J. Marvig, 31055 Toulouse Cedex, France; 3 Center for Advancing Electronics Dresden, TU Dresden, 01062 Dresden, Germany; 4 Dresden Center for Computational Materials Science, TU Dresden, 01062 Dresden, Germany; 5 International Centre for Materials Nanoarchitectonics (MANA), National Institute for Materials Science, 1-1, Namiki, Tsukuba, Ibaraki 305-0044, Japan

## Abstract

Implementing atomic and molecular scale electronic functionalities represents one of the major challenges in current nano-electronic developments. Engineered dangling bond nanostructures on Silicon or Germanium surfaces posses the potential to provide novel routes towards the development of non-conventional electronic circuits. These structures are built by selectively removing hydrogen atoms from an otherwise fully passivated Si(100) or Ge(100) substrate. In this theoretical study, we demonstrate how dangling bond loops can be used to implement different Boolean logic gates. Our approach exploits quantum interference effects in such ring-like structures combined with an appropriate design of the interfacing of the dangling bond system with mesoscopic electrodes. We show how OR, AND, and NOR gates can be realized by tuning either the global symmetry of the system in a multi-terminal setup—by arranging the position of the input and output electrodes—or, alternatively, by selectively applying electrostatic gates in a two-terminal configuration.

Increasing demands from the industry and the public sector have continuously triggered the miniaturization of semiconductor based electronic devices over the past decade towards sub-nm length scales^1^,^2^,^3^. These trends are not only revolutionizing nanoelectronics and related fields, but are also posing a variety of challenges to both the experimental and modelling communities. In particular, issues like tailoring atomic and molecular scale circuit elements, building nanoscale molecular-based logic gates as basic elements to perform computing as well as gaining control over the interconnects between active nanoscale components lie at the forefront of current research efforts^4^,^5^,^6^,^7^,^8^,^9^,^10^. One major issue is also the feasibility to integrate novel, unconventional device components into standard semiconductor technologies.

A physical system, which may in a natural way become viable for integration into conventional semiconductor based electronic architectures, are dangling bond (DB) nanostructures—atomic scale arrangements built on the surface of hydrogen passivated Si(100) or Ge(100) surfaces by removing H atoms with the help of an STM tip^6^. This selective engineering at the atomic scale opens the fascinating possibility to design planar nanocircuits with complex geometry and tunable conduction properties^11^,^12^,^13^,^14^.

Several attempts has been made over the past few years to build in Boolean functions using DB systems^2^,^15^,^16^,^17^,^18^. The first attempt in this direction was based on single atom switching along an atomic scale circuit^19^. The wire is conductive when a switching atom is located in the atomic wire, and is supposed to be non-conductive when this switching atom is moved away from the wire by an electric field. Following this strategy, several groups proposed to use dangling bond states to engineer nanoscale devices^13^,^14^ and ref. 15 discusses further the design rules for switches and logic gates now on atomic scale. This design is based on two principles: surface tunneling effect and the use of electronic destructive interference. Thus, to be able to switch from the ON to the OFF state in an atomic scale switch, one should remove two H-atoms creating a DB T-junction that induces destructive interference, or conversely add two H atoms breaking the DB wire surface conductance. This allows to construct inverter and follower, and by combining them, the logic gates AND, NAND, NOR, and OR. However, a major disadvantage of this approach consists in the necessity of rebuilding the circuit with an STM to be able to operate between the logic states, thus considerably reducing the operational speed. In the second approach tri-naphthylene molecules^5^,^16^ were physisorbed on the Si surface for making contact with DBs wires allowing to realize a semi-classical OR gate. Here, the logic operations are supposed to be performed within the molecule itself, while the DB wires served only as a interconnects to metallic nano-pads.

In this study, we demonstrate several possible alternative strategies to implement Boolean gates by tuning the electrical response of dangling bond loop nanostructures via quantum interference effects. The latter allow to suppress or increase the linear conductance of the system within specific energy windows by promoting destructive or constructive interference. Combining such quantum interference effects with external electrostatic gating and with different arrangements of the nano-electrodes contacting the DB loops allows us to propose different atomic-scale architectures implementing Boolean gates. A major advantage of our proposal lies in the fact that no mechanical manipulation of the circuit (as done with an STM tip) is necessary in order to implement the logic functions.

**Three-terminal setups of silicon dangling bond loops**

Desorption of H-atoms leads to the emergence of electronic states located in Si(100):H surface electronic band gap. the silicon band gap^6^,^20^,^21^,^22^. It is worth noticing that although the dominant spectral contribution to the DB states is clearly provided by the top most depassivated Si surface atoms, non-negligible contributions can also be found up to the 4th–5th silicon layer underneath. As a result, a realistic theoretical description of the electronic structure and charge transport through such structures needs to include these contributions, too^15^,^22^. However, due to the relatively weak coupling to the bulk states, it seems feasible to engineer the DB response to external stimuli and to implement different functionalities as those being at the focus of this investigation. We will focus on structures with two specific types of arrangements of depassivated silicon atoms: zigzag and straight. Based on these two possible arrangements, the dangling bond loops are then built as illustrated in Fig. 1. Clearly, more complex configurations may be built, but loops offer the advantage of being structurally simple, and at the same time, provide a minimal playground to explore quantum interference effects and the implementation of Boolean gates.

To probe the transport properties of the dangling bond nanostructures, electrical nano-contacts need to be engineered. In ref. 22 we showed that graphene nanoribbons provide a good contact conductance to the dangling bond and are mechanically stable junctions to dangling bond structures, while being weakly interacting with the (passivated) silicon surface. We thus have chosen for the demonstration of logic gates graphene nano-ribbons as nano-electrodes. Our results should however not be sensitive to the choice of the electrodes as far as the basic quantum interference features of the dangling bond loops are preserved.

Our first proposed setup is a three-terminal geometry, where three (graphene nanoribbon) electrodes are contacting the DB loop structure. Each nanoribbon has a single terminal carbon atom directly contacting a silicon atom on the loop. As previously shown^22^, single carbon atom contacts provide the best transport pathway in the geometries at the focus of this study. Moreover, apart from the atoms closest to the DB loop, the remaining part of the graphene nanoribbons only weakly interacts with the silicon substrate, so that charge transport is taking place through the dangling bond region.

The parts of the DB loop between the leads might be considered as two DB wires in parallel Fig. 2(a). If these two wires are identical (assuming additionally mirror symmetrical positions of the leads and in weak electronic coupling regime) the total conductance is proportional to the sum of individual conductances plus a quantum mechanical term resulting in constructive interference^23^,^24^: 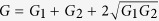. If however the electronic pathways in the clockwise and counterclockwise directions are different (due e.g. to an asymmetric positioning of the leads or to different nearest neighbor electronic couplings in both directions), then destructive interference can set in, similarly to a Mach-Zehnder interferometer. Thus, attaching the leads to different Si atoms on the long sides of the loop provides a way to fine tune both *G*_1_ and *G*_2_ as well as the quantum interference term in the total conductance.

In order to implement an OR logic gate, we study how the lead coupling positions within the loop influence the tunnelling current intensity through it. The goal is to find an optimum for the following situation: the current between the input terminals on one side of the loop and the output one on the other side should be of similar magnitude, while the current between two input terminals on the same side of the loop should be suppressed playing with destructive interferences. This guarantees that there is no parasitic current flowing between the input terminals, which would compromise the implementation of the Boolean gate.

The most natural way to achieve these conditions is to place two nano-electrodes symmetrically as shown in Fig. 2(a) for the terminals labelled A and B. The computed zero-bias quantum mechanical transmission coefficients are plotted in Fig. 2(b), where only the low energy spectral range is shown, where dangling bond states are contributing (energy window ranging from ~ –0.3 – 0.1 eV). First notice that the transmission is very low over almost all the energy window, with the exception of the previously indicated spectral range, where dangling bond states contribute. Thus, this range will give the major contribution to the tunnelling current. Moreover, within this energy window, the zero-bias transmission between terminals A and B is strongly suppressed. The behavior of the transmission is then reflected in the computed *I* − *V* characteristics, as shown in Fig. 2(c): the current between terminals A–C and B–C are similar while that between A and B is strongly suppressed in the applied bias interval where a Boolean gate can be implemented. Applied voltages larger than 0.6 V lead to current leakage through the substrate and therefore bulk states start to contribute. In the bias range highlighted in grey, we can thus assign the “0” logical output to the case of low current and the logical “1” output to the high current situation, using the voltage differences *V*_*AC*_ and *V*_*BC*_ between terminals as the inputs and the resulting total current *I*_*AC*_ + *I*_*BC*_ as an output. Thus, e.g. by fixing the voltage ~0.4 V and assigning logical “1” to currents larger than 90 nA, applying a voltage between electrodes will result in the “1” state. If a voltage is not applied, the device returns to its “0” logical status. This is exactly the logical truth table for the OR gate shown in the inset of Fig. 2(c).

**Electrostatic top gate**

A possible alternative to a three-terminal setup to design a logic gate is the positioning of an electrostatic gate on a two-terminal junction. We have modelled a planar capacitor-like electrostatic gate geometry by applying an external uniform electric field. We consider two cases when an electric field of strength 0.54 V/A is applied perpendicularly to the Si surface and directed towards (“down” direction perpendicular to the loop as presented in Fig. 3) and from (“up” direction) the surface. In Fig. 3 we compare the influence of the field on the electronic DOS (Fig. 3(c,e)) and on the transmission function (Fig. 3(b)) for the straight loop geometry. Once the electric field is applied, it leads to a redistribution of the electronic states as well as to a shift of the corresponding Fermi energies with respect to the system with no external field. When the electric field is pointing from the surface, Fig. 3(c), the Fermi level shifts towards the center of the Si band gap and the states become localized close to the electrode-DB interface as shown in Fig. 3(d). On the contrary, if the electric field is applied in the reversed direction (towards the Si surface) the Fermi energy stays close to the valence band, more states appear close to the Fermi level, and they are delocalized within the DB loop, see Fig. 3(f). These two different responses to the external field result in drastic changes of the transmission function close to *E*_*F*_ as shown in Fig. 3(b). Compared to the case with no applied field, one can see that in the case of field “up” (c)–(d) the transmission is reduced to 2 × 10^−5^ at maximum, while if the applied field has the “down” direction, the transmission spectrum is broader around *E*_*F*_, which means that more DB states contribute to the transport.

The efficiency of the DB loops as a response to the gate voltage is characterized by its transconductance. It can be calculated as the derivative of the current with respect to the applied electrostatic gate potential *dI*/*dV*_gate_; for the DB loops studied here we obtain values in the range of 2.5 × 10^−9^ A/V and up to 6 × 10^−8^ A/V. This is between the *C*_60_ single molecule and single carbon nanotube transistor transconductances^25^. Further details on the DB loop response to the external electric field and its transconductance properties are provided in the Supplementary Information.

The interplay between quantum interference through the loop and the influence of the electrostatic gate results in tunable transport properties of the DB system. Larger transconductance values for the straight DB loop can be explained due to the asymmetric DOS spectra around *E*_*F*_, while zigzag loops display a more symmetric profile^22^. Hence, the most illustrative example is a case of an asymmetric arrangement of the left and right electrodes to a straight DB loop. If there is no electric field applied, destructive interference results in poor transmission. However, under the applied electric field a redistribution of the charge density can take place within the DB loop, the Fermi level can be shifted, and this can lead to a switching between constructive and destructive interference as shown in Fig. 4. We can now use the applied bias voltage between terminals B and C and the applied electrostatic field as input variables to implement an “AND” logic gate. Similar to the previous “OR” logic gate we can assume low current results in logical “0” and high current realizes the logical “1”.

If the electric field is zero, only a very low current flows through the junction at bias up to 0.2 V due to destructive interference effects^22^. On the contrary, by applying the gate voltage, a strong increase of the current takes place already for *V* > 50 mV and the two currents can differ by about a factor of up to 6–7 at *V* ~ 0.4 V. Thus, for almost the entire bias range the proposed setup can act as an AND Boolean gate. The corresponding truth table is shown on Fig. 4(b). The large difference in the current values with and without the gate makes the logical states well distinguishable.

Other kind of logic gates can be designed by combining the previous two setups. As an example, we show in Fig. 5(a) a pair of straight DB loops combined in series. As previously mentioned, the applied electric field in the “up” direction significantly reduces the current through the system. The same hold for the two DB loops connected in series without any phase loss in between the two loops. We assume that the electric field might be applied locally, i.e. on one part of the system only. In this way we can switch between conducting and non-conducting states of the left (blue) and right (red) loops independently. This is of course an ideal situation, which may be difficult to realize experimentally, at least for loops with small sizes. However, our goal here is to illustrate the potential richness of dangling bond architectures to implement Boolean functions. Applying the field on one loop only results in low transmission of the whole system, since the two loops are connected in series, see Fig. 5(b), which brings it to the low current regime as shown in Fig. 5(c). If the field is applied on the whole system it yields a similar low current as in the previous case. Therefore, one can build one of the universal logic gates—a NOR gate—, where the applied electric fields *E*_1_ and *E*_2_ may serve as the inputs, and the resulting current between the leads as the output. If no field is applied the current reaches high values of ~300 nA around a bias voltage of 0.3 V (the voltage range where the two DB loops in series can work as a NOR gate is highlighted in grey in Fig. 5(c)), which might serve as logical “1”. If the field is applied on any DB loop separately or on both loops together, it restricts the electron flow between the electrodes and results in the logical “0” state.

## Discussion

**Figure 2:**
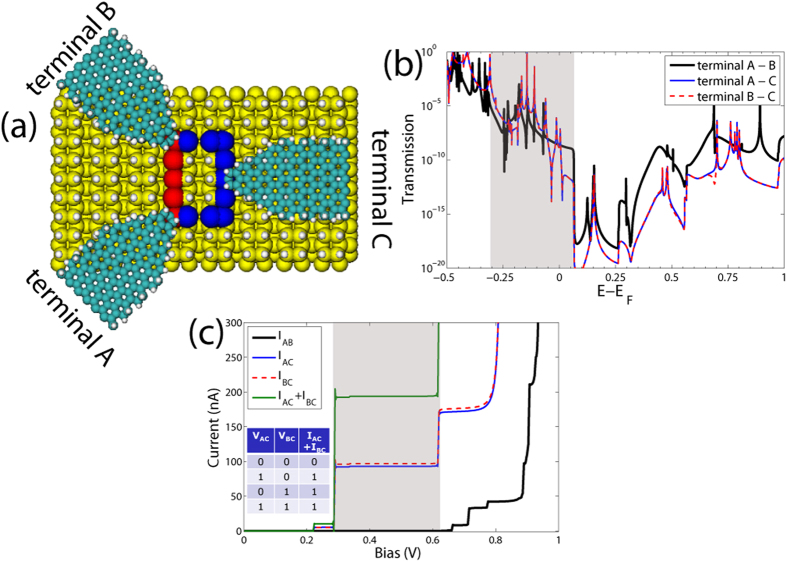
(**a**) Three-terminal setup built with carbon nanoribbon electrodes and a straight DB loop to implement an OR gate. DBs are highlighted with blue and red color to show different electron paths between the electrodes. (**b**) Energy dependent quantum mechanical transmission function: within the energy window (shaded in grey), where the setup can work as a logic gate, the A-to-C and B-to-C transmissions are almost identical due to the symmetric arrangement of the three electrodes, while the A-to-B transmission is strongly suppressed due to interference effects. (**c**) *I* − *V* characteristics, when a voltage is applied between terminals A and B (black curve), A and C (blue solid curve), and B and C (red doted curve), and their sum (green curve). The truth table for the OR gate is shown on the inset. The working bias windos for the OR gate is shaded in grey.

We have shown in this study different strategies to implement Boolean functions by exploiting quantum interference through diverse silicon dangling bond loops. Three-terminal setups and two-terminal ones with an applied electrostatic gate were addressed as well as a series arrangement of two dangling bond loops. We showed that by specific choices of the contact position of the electrodes along the loop, quantum interference effects can be fine tuned and can be exploited to implement an OR logic gate in a three-terminal junction (Fig. 2).

**Figure 4:**
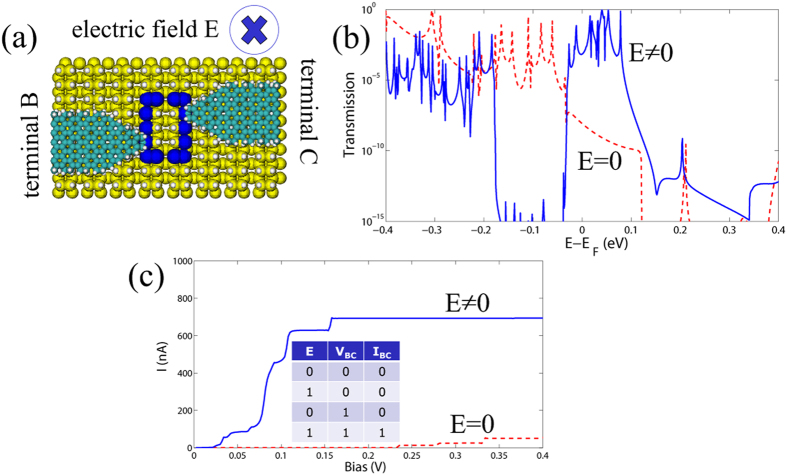
(**a**) Two terminal setup made with carbon nanoribbon electrodes and a straight DB loop under an applied uniform external electric field directed towards the Si surface (“down” in Fig. 3). The electrodes B and C are connected asymmetrically to the loop. (**b**) *I* − *V* characteristics with the voltage applied between B and C terminals and with zero electric field (red dotted line) and an electric filed of magnitude 0.54 *V*/Å (blue solid line). The truth table for the AND logic gate is shown in the inset. (**c**) Transmission function between terminals B and C with zero electric field (red dotted line) and under the applied field (blue solid line). Notice the strong suppression of the transmission by the applied field.

Alternatively, keeping a two-terminal setup, but adding an electrostatic gate allowed to realize an AND logic gate (Fig. 4). Starting with an asymmetric arrangement of the nanoribbon electrodes, which provided a low transmission around the Fermi energy as a result of destructive interference, an applied field induced a shift in the position of the transmission resonances in such a way that the corresponding electric currents (with and without the field) differed by about one order of magnitude. We showed that depending on the direction of the applied electric field the transport properties can be suppressed or enhanced and the ratio between logical “0” and “1” outputs reaches few orders of magnitude, that makes them well distinguishable.

Other types of logic gates can be built based on the design rules suggested in this paper. For example, knowing the response to the electric field of the DB systems, one can use the classical principles of transistor based circuits, where the logic gates are built with several transistors and controlled by the applied base-emitter and base-collector voltages^26^. Additional DB configurations such as T-junctions could be investigated to further control quantum interference^15^ under an applied electric field. This approach might be be extended to a fully planar design, where instead of a top gate, the voltage is applied between planar electrodes made with gold nanopads superimposed on the passivated Si surface^8^,^15^,^16^. Another possibility would exploit time-dependent perturbations: interference patterns under an applied AC field/voltage or effects of charging/discharging of the DB dimers^14^,^27^ on the transport properties are still open issues. In addition, planar DB based systems might be efficiently controlled by modifying the Si surface with organic molecules that undergo reversible conformational changes under UV-light, visible light^28^,^29^, or under the action of voltage pulses by an STM tip^30^,^31^.

## Methods

The structures were relaxed with the DFTB code^32^,^33^ using the conjugate-gradient method with an accuracy up to 10^−7^ eV/Å and self-consistent charge calculations (SCC) were carried out with an accuracy of up to 10^−5^e. For each position of the electrodes and each value of the applied electric field SCC calculations and geometry relaxation were carried. The silicon surface was modelled using periodic boundary conditions. The DFTB approach allows to efficiently treat large structures (up to 2000 atoms) due to preparametrized Slater-Koster tight binding model. Charge transport calculations were carried out by using the Hamiltonian and Overlap matrices generated by the DFTB calculations. The energy dependent quantum mechanical transmission function *T*_*ij*_(*E*) between terminals *i* and *j* was computed using the Landauer-Büttiker formalism^34^: 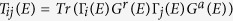. Here, 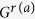 are retarded (advanced) Green’s function of the system, that describes central DB system taking into the account the interaction with the leads by means of the self-energy function 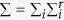, 
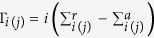
 is the electrodes spectral density. *T*_*ij*_(*E*) describes the probability of electrons with energy *E* entering to the system from lead *i* with the retarded(advanced) self-energy 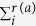 to be transmitted to the lead *j* with the correspondent self-energies 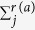. These self-energies are calculated for each leads using the iterative Lopez-Sancho procedure^35^. Within this procedure we take nanoribbon slices at a distance of few dimer rows away from the central part, that includes DB surface atoms, Si surface underneath and the nanoribbon tips. These together with periodic boundary conditions, nanoribbon relaxation procedure and contribution of the deeper silicon layers to the surface DB state results in the large spatial dimensions of the Si system we consider: 8 substrate atoms, 9 atoms wide and 8 dimer rows long in the transport direction. The current between terminal *i* and *j* is computed as 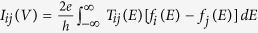, here *T*_*ij*_(*E*) is the zero bias transmission, and *f*_*i*(*j*)_(*E*) are the Fermi function of electrode *i* and *j* respectively.

## Additional Information

**How to cite this article**: Kleshchonok, A. *et al.* Quantum interference based Boolean gates in dangling bond loops on Si(100):H surfaces. *Sci. Rep.* **5**, 14136; doi: 10.1038/srep14136 (2015).

## Supplementary Material

Supplementary Information

## Figures and Tables

**Figure 1:**
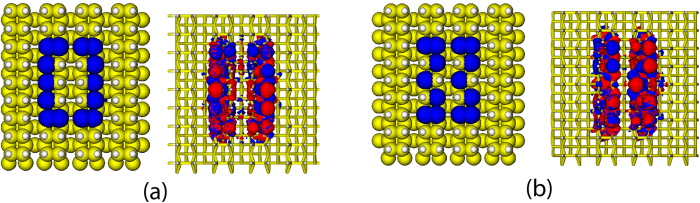
(**a**) Straight and (**b**) zigzag DB loops. Left panels of (**a**) and (**b**) show the H-passivated Si(100)(2 × 1) surface, while depassivated atoms are highlighted in blue. The corresponding right panels of a) and b) display top views of the real part of the electronic wave function of the localized DB states, where red color corresponds to the positive and blue to the negative contributions.

**Figure 3:**
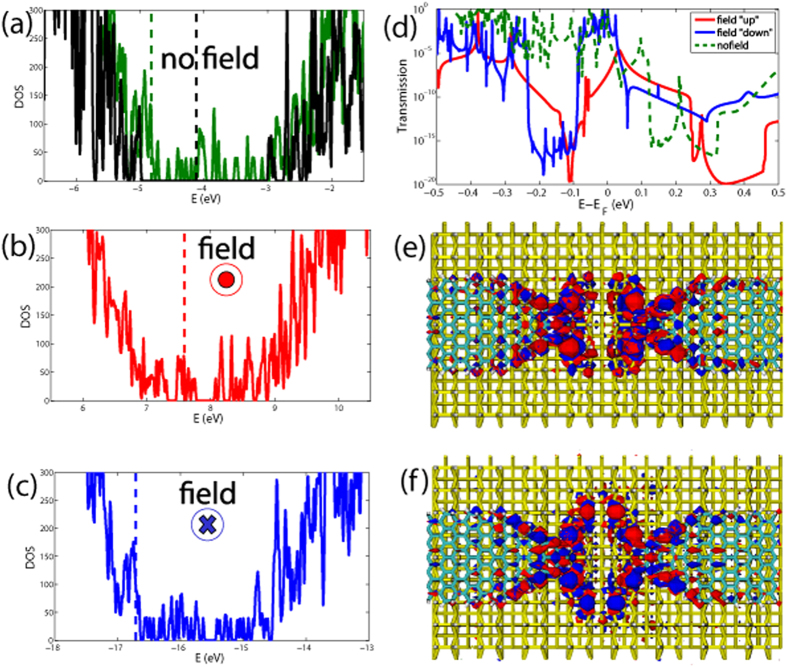
(**a**) Electronic density of the states (DOS) of the fully passivated Si surface (black) and DB straight loop (green), (**b**,**c**) the DB loop under an applied electric field perpendicular to the surface and pointing “up” (out of the surface, panel (**b**)), and “down” (into the surface, panel (**c**)). The corresponding positions of the Fermi levels in all cases are indicated by the vertical dotted lines. (**d**) Transmission function for zero applied gate field and for the two possible directions of the applied field. Notice the strong difference in the transmission around the Fermi energy for both field directions. (**c**) DOS of the straight loop under the applied electric field of intensity 0.01 a.u. Panels (**e**,**f**) show the real part of electron wave function within the energy window 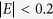 eV around the Fermi energy for the “up” (**e**) and “down” (**f**) field directions, where red color corresponds to the positive and blue - to the negative contributions.

**Figure 5:**
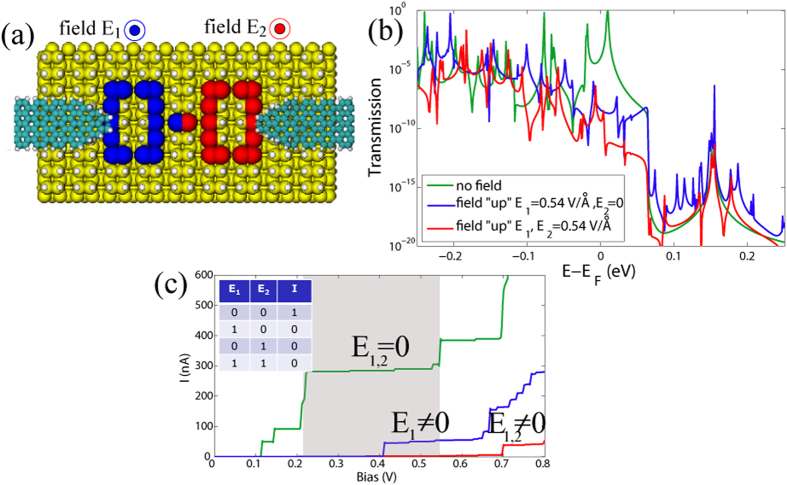
(**a**) Two terminal setup made with carbon nanoribbon electrodes and two straight DB loops in series under an applied uniform electric field directed “up” from Si surface. *E*_1_ is applied on the left loop, denoted with blue color, and *E*_2_ on the right loop (red color). (**b**) Transmission function for the cases with zero field (blue curve), *E*_1_ = 0.01 a.u., *E*_2_ = 0 (magenta curve), and *E*_1_ = *E*_2_ = 0.01 (red curve). (**c**) *I* − *V* characteristics of the two-terminal device for zero field (blue curve), and for the cases *E*_1_ = 0.01 a.u., *E*_2_ = 0 (magenta curve), and *E*_1_ = *E*_2_ = 0.01 (red curve).The relevant voltage range for implementing the NOR gate is highlighted in grey and the inset shows the corresponding truth table.
