# Supplementary material for: Quantum interference based Boolean gates in dangling bond loops on Si(100):H surfaces
Source: Sci Rep. 2015 Sep 15;5:14136. doi: 10.1038/srep14136 (PMC4642695; doi:10.1038/srep14136)
Supplement: Supplementary Information [file srep14136-s1.pdf]

Supplementary information

Andrii Kleshchonok[1,*], Rafael Gutierrez[1], Christian Joachim[2,5], and Gianaurelio Cuniberti[1,3,4]

[1]Institute for Materials Science,Dresden University of Technology, TU Dresden, 01062 Dresden, Germany
[2]GNS & MANA Satellite, CEMES-CNRS, 29 rue J. Marvig, 31055 Toulouse Cedex, France
[3]Center for Advancing Electronics Dresden, TU Dresden, 01062 Dresden, Germany
[4]Dresden Center for Computational Materials Science, TU Dresden, 01062 Dresden, Germany
[5]International Centre for Materials Nanoarchitectonics (MANA), National Institute for Materials Science, 1-1, Namiki, Tsukuba, Ibaraki 305-0044, Japan
[*]andrii.kleshchonok@nano.tu-dresden.de

ABSTRACT

We provide details on the loops response to the external electric fields and their transconductance properties.

Transconductance of the dangling bond loops. We performed a systematic study of the influence of the electrostatic gating on the transport properties of DB loops. For this we calculated $I-V$ characteristics for different topologies of the loops and symmetrical and asymmetrical connection of the leads. We considered fields up to 0.015 a.u. (0.816 V/Å); since the DB states are isolated and up to few tens of meV wide we do not need large fields in order to operate between electronic states. At these values of the field intensity we do not see large changes in the atomic structure of the DB system upon relaxation. As mentioned in the main text, different directions of the electric field can suppress or enhance the transmission. Therefore, we studied two different configurations: (i) symmetrical lead configuration Fig. S1(a),(c) under the field directed "up" in order to switch off the current within the loop and the inverse situation (ii), when the leads are connected asymmetrically and the electric field is pointing "down" (see Fig. S1(b),(d)) induce current within the loop. Fixing the bias voltage at 0.2 V for each configuration we scan over different field intensities, this allows us to estimate the transconductance dI/dE_{gate}: (a)-$-4.99 \cdot 10^4$ nA/a.u., (b)- $3.27 \cdot 10^5$ nA/a.u. , (c) - $-1.05 \cdot 10^4$ nA/a.u., (d)- $1.54 \cdot 10^5$ nA/a.u. Assuming that the electric field is induced by two parallel infinite plates with the distance between them of 10 nm we can obtain the transconductance in terms of the gate voltage dI/dV_{gate} in the range between $2.5 \cdot 10^{-9}$ A/V up to $6 \cdot 10^{-8}$ A/V.

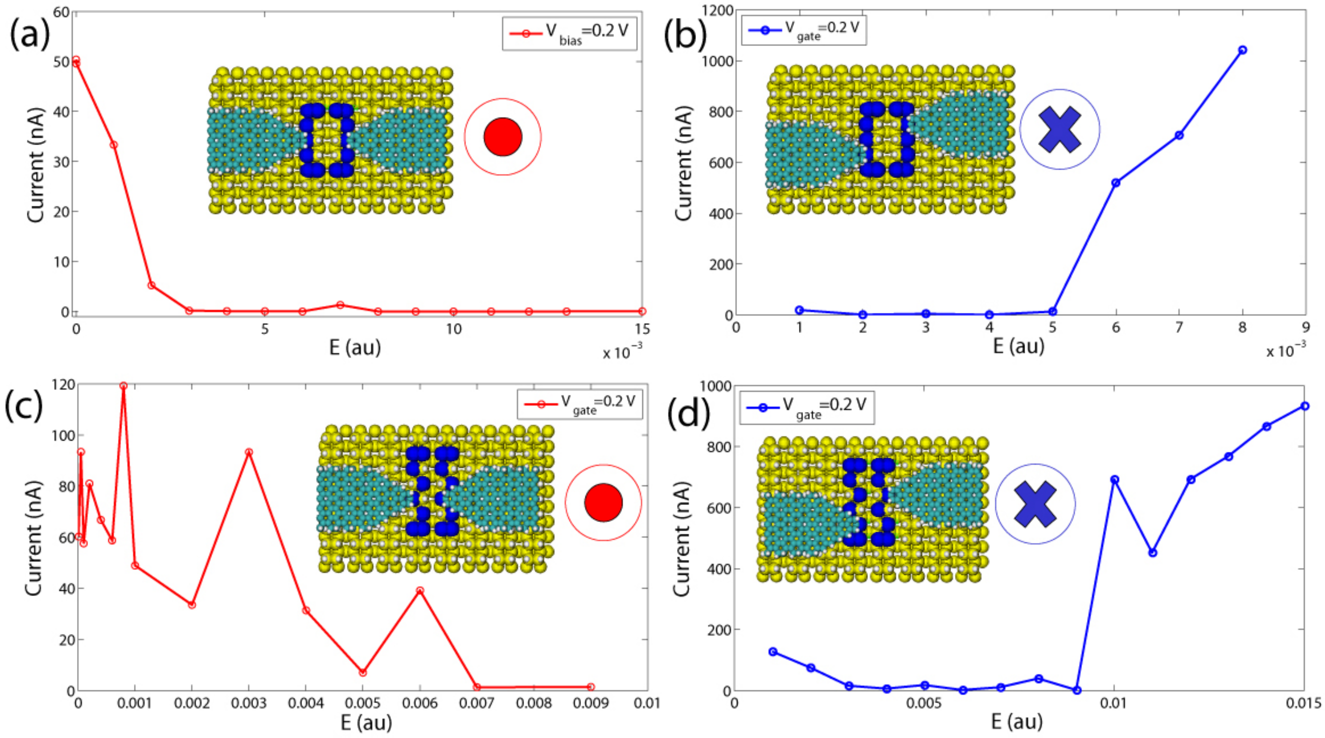

Figure S1. Current as a function of the applied electric field at fixed bias voltage of 0.2 V for (a) straight DB loop with symmetric coupling of the leads; (b) straight DB loop with asymmetric coupling of the leads; (c) zigzag DB loop with symmetric coupling of the leads; (d) zigzag DB loop with symmetric coupling of the leads. In (a) and (c) the electric field is directed away from the Si surface and current is suppressed, while in (b) and (d) the electric field is directed to the Si surface and current is enhanced.
